# Safety of pull-type and introducer percutaneous endoscopic gastrostomy tubes in oncology patients: a retrospective analysis

**DOI:** 10.1186/1471-230X-11-23

**Published:** 2011-03-16

**Authors:** Evi Van Dyck, Elisabeth J Macken, Bernard Roth, Paul A Pelckmans, Tom G Moreels

**Affiliations:** 1 Division of Gastroenterology & Hepatology, Antwerp University Hospital, Wilrijkstraat 10, B-2650 Antwerp, Belgium

## Abstract

**Background:**

Percutaneous endoscopic gastrostomy (PEG) allows long-term tube feeding. Safety of pull-type and introducer PEG placement in oncology patients with head/neck or oesophageal malignancies is unknown.

**Methods:**

Retrospective analysis of 299 patients undergoing PEG tube placement between January 2006 and December 2008 revealed 57 oncology patients. All patients with head/neck or oesophageal malignancy were treated with chemo- and radiotherapy. In case of high-grade stenosis introducer Freka^® ^Pexact PEG tube was placed (n = 24) and in all other patients (n = 33) conventional pull-type PEG tube. Short-term complications and mortality rates were compared.

**Results:**

Patients' characteristics and clinical status were comparable in both groups. Short-term complications were encountered in 11/24 (48%) introducer PEG patients as compared to only 4/33 (12%) pull-type PEG patients (P < 0.05). Accidental removal of the introducer PEG tube occurred in 4/24 (17%) with need for surgical intervention in 1 *vs*. 0/33 (0%, P < 0.05). Wound infection occurred in 3/24 (12%) leading to septic shock and admission to intensive care unit (ICU) in 1 *vs*. 3/33 (9%, NS). Finally, 3/24 gastrointestinal perforations (12%) resulted from a difficult placement procedure *vs*. 1/33 (3%), leading to urgent surgical intervention and admission to ICU. Two introducer PEG patients died at ICU, resulting in an overall mortality rate of 8% *vs*. 0% (P = 0.091).

**Conclusion:**

The introducer Freka^® ^Pexact PEG procedure for long-term tube feeding may lead to significantly higher complication and mortality rates in patients with head/neck or oesophageal malignancies treated with chemo- and radiotherapy. It is suggested to use the conventional pull-type PEG tube placement in this group of patients, if possible.

## Background

Percutaneous endoscopic gastrostomy (PEG) was first performed in 1979 using the pull-type technique [1]. Since then feeding tubes have been adapted, but the pull-type technique is still the standard procedure for endoscopic PEG placement [2]. It allows longterm tube feeding, when oral feeding is not possible, or when extra feeding is necessary [3]. PEG placement involves an upper gastrointestinal (GI) endoscopy, usually under conscious sedation and with the use of local anesthesia at the gastrostomy site. Prophylactic use of antibiotics is advisable [4]. After inflation of air into the stomach in order to obtain maximal apposition of the gastric and abdominal walls, the gastrostomy site is located based on the combination of light transillumination and finger indentation of the abdominal wall. In the pull-type procedure the feeding tube is pulled through the mouth into the stomach and through the abdominal wall. However, in several clinical situations the classical pull-type PEG procedure is not possible or contraindicated. In case of high-grade stenosis caused by an oesophageal tumor or a head and neck tumor, a conventional upper GI endoscopy may not be possible or the internal bumper of the PEG-tube may not pass. Also, the risk of entmetastases at the site of the gastrostomy is real [5]. Finally, high volume ascites in the abdominal cavity is also a contraindication for a pull-type PEG procedure because of the risk of leakage [6,7].

**Figure 1:**
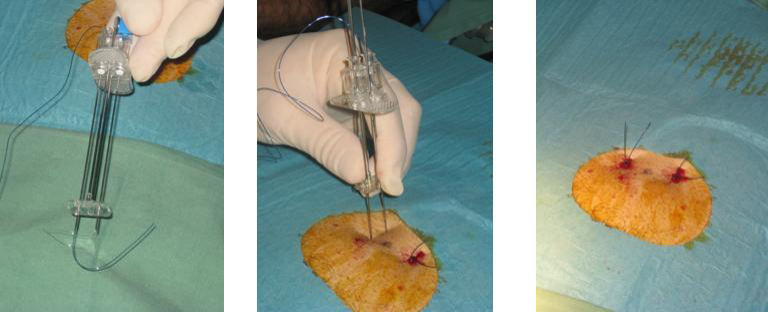
**Freka^® ^Pexact procedure showing the double needle to create a 2-suture gastropexy**.

**Figure 2:**
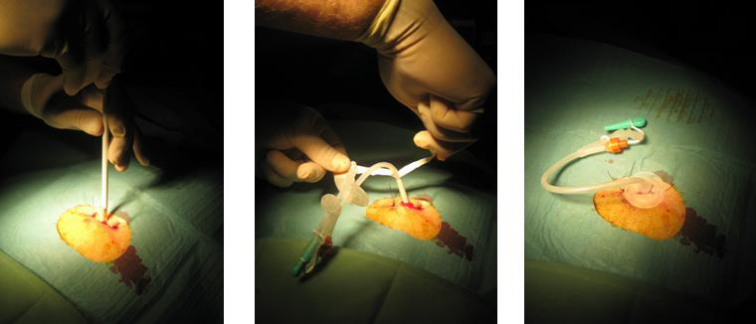
**Freka^® ^Pexact procedure showing introduction of the trocar with the peal-away sheet and final position of the temporary PEG tube**.

These limitations of the conventional pull-type PEG led to the development of the introducer PEG, with or without the combination of a gastropexy [6-9]. After filling the stomach with air and locating the site of puncture by means of light transillumination and finger indentation, 2 or 4 sutures are applied under endoscopic guidance using a specifically designed introducer needle, resulting in a gastropexy of the anterior gastric wall to the ventral abdominal wall (Figure 1). Next, a trocar with a peal-away sheet is introduced through the abdominal wall into the stomach (Figure 2). Alternatively, a guidewire is introduced via the Seldinger technique and the peal-away sheet is introduced after progressive dilation of the gastrostomy. Finally the feeding tube is progressed through the sheet which is then pealed off. An inflatable balloon at the tip of the feeding tube serves as internal bumper. Depending on the technique, the feeding tube is either temporary or definitive. This technique can be used in case of high grade stenosis since it allows transnasal endoscopy using an ultra thin endoscope, even without sedation [10]. Thanks to the gastropexy, this technique can also be safely used in case of ascites [6].

Patients with oesophageal or head and neck malignant tumors may need tube feeding because of poor oral food intake due to malignant stenosis or due to radiation- and chemotherapy-induced oesophagitis [11]. Often PEG-tube feeding is mandatory. In these cases one may argue that the introducer PEG tube may serve better. However, little is known on the outcome of introducer PEG tubes in these patients. This study retrospectively analyzed the outcome and short-term complications of both pull-type and introducer PEG tubes in oncology patients with oesophageal or head and neck malignancies.

## Methods

All medical records were reviewed of patients who underwent PEG tube placement at the Antwerp University Hospital Endoscopy Unit from January 2006 until December 2008. The local ethical committee of the Antwerp University approved the retrospective analysis of the medical files. All patients or their relatives provided informed consent for the PEG procedure. In total 299 procedures were performed. All oncology patients with oesophageal or head and neck malignancies were selected. Indication for PEG tube feeding was made by the treating oncologist. PEG tube was placed 1 day before the start of combined radiotherapy and chemotherapy in all cases. Introducer PEG placement with gastropexy (Freka^® ^Pexact Fr 15, Fresenius Kabi AG, Bad Homburg, Germany) was only performed in case of high-grade malignant stenosis, rendering the use of a conventional upper GI endoscope difficult or impossible. The decision to choose between pull-type and introducer PEG was at the discretion of the endoscopist. The introducer PEG procedure is shown in Figures 1 and 2. Whenever possible, conventional pull-type PEG (Freka^® ^PEG-Set Gastric Fr 15 & 20, Fresenius Kabi AG, Bad Homburg, Germany and Flocare PEG Set Fr 18, Nutricia, Schiphol Airport, The Netherlands) placement was performed. All procedures were performed by staff members in collaboration with trainees. They were assisted by an endoscopy nurse during the procedure. Finally, results of the pull-type and push-type PEG procedures were compared in the selected group of oncology patients. Data on patients' age, sex, serum albumin, WHO performance status, sedation and short term complications (within 8 weeks after PEG placement) were retrospectively collected and were statistically analyzed using unpaired Student's t test in case of continuous variables and Mann-Withney test in case of discontinuous variables and Chi square test for contingency table statistics. P < 0.05 was considered statistically significant. Results of continuous variables are expressed as mean ± SEM (standard error of the mean) and results of discontinuous variables as median with 25^th ^and 75^th ^percentile. The WHO (World Health Organization) performance status is a scale for assessing general health, ranging from 0 (healthy) to 5 (death) [12].

## Results

Of the 299 PEG procedures performed, 57 involved oncology patients with oesophageal (n = 9) or head and neck malignant tumors (n = 48). All procedures were performed under conscious sedation using midazolam, administered according to the patients' needs. Linisol 2% was used for local anesthesia of the abdominal wall. All patients received intravenous antibiotic prophylaxis with either amoxiciline-clavulanic acid, levofloxacine or cefacidal, except for 3 patients who were under antibiotic treatment at the time of PEG placement. Antibiotic use was dependent on the patient's known allergies. Pull-type PEG was placed in 33 patients (58%) and introducer PEG in 24 (42%). Table 1 shows that both groups were comparable regarding patient characteristics like age, sex and clinical status, expressed in serum albumin levels and WHO performance status. All patients underwent combined radiotherapy and chemotherapy, starting at least 24 hours after PEG placement. Retrospective analysis of the medical records revealed several types of short-term complications: local infection of the skin, prolonged bleeding of the gastrostomy, perforation of the GI tract, accidental removal of the feeding tube, need for urgent surgery and death. Overall, 4 (12%) complications were encountered in the pull-type PEG group without mortality, whereas a total of 11 (48%, P < 0.05) complications were seen in the introducer PEG group (Table 2). In the remaining 242 patients, 24 (9.5%) complications were registered. Local infection of the gastrostomy was encountered in both groups and was treated conservatively with local disinfection and systemic antibiotic treatment. During the study period, perforation of the GI tract was encountered in both groups. One pull-type PEG tube perforated the sigmoid colon needing surgical intervention. This was an example of interposed colon due to inadequate location of the gastrostomy site by means of transillumination and finger indentation. In the introducer PEG group, we observed 3 perforations and 2 of them had severe clinical consequences leading to urgent surgery in 2. One was only a shortlasting episode of pneumoperitoneum with mild abdominal discomfort without further clinical significance. It is supposed to result from inadequate insertion of the double needle with one inside the stomach and the other one along the gastric wall. Two patients needed surgical intervention due to overt clinical signs of perforation. The first occurred after accidental dislocation of the tube into the peritoneal cavity shortly after the placement. During rescue surgery, a laceration of the insertion site in the gastric wall was seen. The second was noticed because of leakage of the tube feeding from the gastrostomy site and abdominal pain. Rescue surgery also showed a laceration of the gastric wall but with the feeding tube still in place. Unfortunately, both patients died at ICU shortly after due to respiratory complications. Although statistical analysis revealed no significant difference in mortality rates (0% versus 8%, P = 0.091), the 2 deaths after urgent rescue surgery are considered major drawbacks of the introducer PEG procedure.

**Table 1:** Patients' characteristics.

	Pull-type PEG	Introducer PEG	
number patients (%)	33 (58)	24 (42)	
male/female (%)	27/6 (82/18)	18/6 (75/25)	P = 0.533
mean age ± SEM (years)	65 ± 2	64 ± 3	P = 0.774
mean serum albumin ± SEM (mg/dl)	3.2 ± 0.1	3.0 ± 0.1	P = 0.130
median WHO performance (25^th ^- 75^th ^percentile)	1 (0-3)	2 (1-3)	P = 0.212

SEM: standard error of the mean

WHO: World Health Organization performance status

P < 0.05: P value from statistical analysis (Unpaired Student's *t *test for continuous variables and Mann-Whitney test for discontinuous variables, Chi square test for contingency table statistics).

**Table 2:** Comparison of complications between Pull-type and Introducer PEG.

Complications	Pull-type PEG	Introducer PEG	
total (%)	4 (12)	11 (48)	P = 0.004
			
local infection (%)	3 (9)	3 (12)	P = 0.678
bleeding (%)	0 (0)	1 (4)	P = 0.237
perforation (%)	1 (3)	3 (12)	P = 0.167
tube removal (%)	0 (0)	4 (17)	P = 0.015
			
surgery (%)	1(3)	3 (12)	P = 0.167
mortality (%)	0 (0)	2 (8)	P = 0.091

P < 0.05 unpaired Student's *t *test.

In the introducer PEG group, 1 prolonged bleeding of the skin insertion site was observed. The bleeding lasted for a couple of hours and was treated conservatively by local pressure application. In addition, 1 patient from the introducer PEG group needed ICU admission because of sepsis. During chemotherapy he developed neutropenic fever and sepsis caused by a local infection at the insertion site and the gastropexy sutures. Culture showed *Candida species *both at the gastrostomy site and in the blood. The PEG tube needed to be removed.

Finally, accidental removal of the PEG tube within 8 weeks after placement was not seen in the pull-type PEG group, whereas 4 patients (17%, P < 0.05) lost their temporary feeding tube in the introducer PEG group. In 1 patient partial tube dislocation resulted in accidental tube feeding into the peritoneal cavity with severe peritonitis leading to urgent surgery, as described above.

Over the 3 years period complication rates of the introducer PEG tube were randomly divided, without correlation with time and endoscopist (data not shown).

## Discussion

PEG tube feeding is the preferred method to provide long-term tube feeding and its use is widespread nowadays [2]. However, PEG tube placement is an invasive endoscopic procedure with an inherent risk of complications [13]. Major complications resulting from PEG tube placement include peritonitis, hemorrhage, airway aspiration, peristomal wound infection, buried bumper symdrome, and gastrocolic fistula [3,14]. In the literature, complication rates of the conventional pull-type PEG procedure vary from 1% to 30% [3,13-16]. Overall complication rate of pull-type PEG tube placement was 9.5% in non-oncology patients in our retrospective analysis. In oncology patients with head and neck tumors, the complication and mortality rates appear higher, probably related to concomitant treatment with chemotherapy and radiotherapy, reducing the patients' immunity [17]. In this respect, it is noticed that in our center oncologists start chemo- and radiotherapy 1 day after the pull-type and introducer PEG procedures. For safety reasons, one could argue that this immune suppressive treatment should be postponed for 3 to 4 days after PEG placement. However, the therapeutic strategy was similar in both PEG procedure groups. Therefore, the difference in complication rate between the two methods cannot be accounted to the short interval between PEG procedure and start of chemo- and radiotherapy.

Patients with oesophageal or head and neck tumors encompass a specific group of patients often requiring PEG tube placement because of poor oral food intake which may be related to the disease and/or its treatment. Conventional pull-type PEG tube placement is common in these patients. However, high-grade stenosis may impede the introduction of a conventional upper GI endoscope or the passage of the internal bumper of the PEG tube. Moreover, there is a risk of entmetastases at the gastrostomy when the feeding tube is pulled across the malignant tumor through the stomach and the abdominal wall [5]. Therefore, the introducer PEG tube technique was developed and further adapted with an endoscopically controlled gastropexy [6-9]. We used the commercially available Freka^® ^Pexact PEG from Fresenius Kabi AG. Initial data on its use and short-term safety show low complications rates of less than 3% and no mortality [6,9,18,19].

In contrast to the earlier reports, the present retrospective study reveals significant complication (48%) and mortality (8%) rates within 8 weeks after introducer PEG tube placement using the Freka^® ^Pexact in oncology patients with oesophageal or head and neck malignant tumors. After reviewing the patients' medical files and after discussion with the referring oncologists and ear-nose-throat (ENT) specialists, both procedure-related and patient-related complications were encountered.

The double needle from the Freka^® ^Pexact procedure to perform the gastropexy, must be introduced simultaneously into the stomach. However, inadequate introduction may lead to gastric perforation away from the gastrostomy when one of the needles glides along the outer gastric wall. Apparently, this complication does not seem to be related to lack of experience since no change in complication rate was observed over the 3 year period. The use of a single needle with T-bar sutures to create the gastropexy may carry less risk of inadequate introduction into the stomach. The use of non-absorbable sutures may also represent an origin of complication. If not removed appropriately, they may increase the risk of local skin infection, especially in immunocompromised patients under combined radio- and chemotherapy. According to the manufacturer's guidelines, the sutures have to be removed 10 days after gastropexy was performed. And although this was always clearly marked in the follow-up section of the endoscopy report, this was often forgotten by the referring oncologist. In combination with the patients' reduced immunity due to chemotherapy, remaining sutures may lead to local skin infections. Initial reports show lower infections rates with the introducer PEG, probabely because the tube does not pass the oral cavity [19]. In non-immunocompromised patients, the use of prophylactic antibiotics may even be not mandatory [19]. Our results still advocate a stringent follow-up together with the oncologist of the gastropexy and gastrostomy site and the use of antibiotics in immunocompromised patients. The use of absorbable sutures may also limit local skin infections. Finally, according to the manufacturer's guidelines, the temporary PEG tube of the Freka^® ^Pexact procedure needs to be replaced by a definite PEG tube after 4 weeks, due to the weakness of the internal balloon. Although this was also covered in the follow-up section of the endoscopy report, in-time replacement of the temporary PEG tube was also often forgotten by the referring oncologist, leading to accidental tube dislocation and even intraperitoneal tube feeding. Immediate placement of a definite feeding tube is advocated to decrease the risk of accidental tube dislocation.

These results show that some of the complications encountered were probably avoidable with a more stringent follow-up of the gastropexy and gastrostomy in these oncology patients. However, in these patients the endoscopist is only involved in the actual placement of the PEG tube, whereas general follow-up of the patient is done by the oncologist who is focused on the medical treatment of the underlying malignant disease. Apparently, in this group of immunocompromised patients this introducer PEG tube needs more attention as compared to the classical pull-type PEG tube. Better instructions to the referring oncologist and better follow-up by the endoscopist performing the PEG tube placement seems mandatory.

Alternatives to endoscopy to perform gastrostomy with tube feeding are radiological (fluoroscopy- or ultrasound-guided) placement or surgical placement with a mini-laparotomy [20,21]. The method of choice to perform gastrostomy for tube feeding depends on the patient's clinical condition and comorbidities and the physician's experience and preference [22]. At the Antwerp University Hospital endoscopic placement is the preferred method, followed by surgical placement. Radiological gastrostomy is not available.

## Conclusion

The present retrospective study shows that severe short-term complications may occur in patients with oesophageal or head and neck tumors after placement of the Freka^® ^Pexact introducer PEG tube, leading to urgent surgical intervention and even death in a substantial amount of patients. Both procedure-related and patient-related risk factors were identified. Better follow-up of PEG tube daily care is mandatory. Also improvements of the introducer PEG tube and alternatives to PEG are suggested. Adaptations of this push-type PEG procedure are under investigation [23]. Our results suggest that for this specific group of patients the conventional pull-type PEG is still the preferred technique, if possible.

## List of abbreviations

ENT: ear-nose-throat; GI: gastrointestinal; ICU: intensive care unit; NS: not significant; PEG: percutaneous endoscopic gastrostomy; SEM: standard error of the mean; WHO: World Health Organization

## Competing interests

The authors declare that they have no competing interests.

## Authors' contributions

EVD performed acquisition and analysis of data and wrote the manuscript; EJM and BR performed PEG tube placement and revised the manuscript; PAP revised the manuscript; TGM performed analysis and interpretation of data and wrote the manuscript. All authors read and approved the final manuscript.

## Pre-publication history

The pre-publication history for this paper can be accessed here:

http://www.biomedcentral.com/1471-230X/11/23/prepub
